# The Nursing Supplement

**Published:** 1888-05-12

**Authors:** 


### The Nursing Mirror.

All Contributions for this Supplement should be addressed "The Nursing Mirror," care of The Editor, 38, Old Jewry, E.C.

### En Passant.

HOMES IN THE HILLS.—On the 1st of May a Home for Lady Nurses now working in the Punjab was opened at Murree. It is under the charge of a lady superintendent and two nursing sisters belonging to the St. Deny's Sisterhood, Warminster. These ladies will have to look after such officers as fall sick and need nursing, and also work in the Soldiers' Hospital when they can find spare time. Similar homes will shortly be opened at Kasauli and Wellington, for the nurses working respectively in Umballa and Madras.

MEDICAL WOMEN.—St. John's Hospital for Diseases of the Skin has followed the example of the dental hospitals, and thrown open its doors to lady students. They are to atend the lectures in common with the ordinary students, but special arrangements have been made for their clinical instruction. After long struggling and patient waiting, all the obstacles which once barred women's progress in the profession of medicine, are gradually being broken away.

LEWES INFIRMARY.—The contributions towards a testimonial for the retiring matron of the Lewes Infirmary have already reached nearly £50. The subscription list is headed by the Mayor of Lewes. Mrs. Webb is deeply grateful for the kindness shown her, and the effort now being made on her behalf. Once the Pension Fund is started, such appeals as the above will become a thing of the past.

VACCINATION.—There was a letter in a medical contemporary last week called "Our Unprotected Nurses," and proclaiming that, though an epidemic of small-pox is expected, none of our nurses are seeking protection from vaccination. The writer is unwittingly unjust, for in at least two of the largest metropolitan hospitals, the nurses have all been questioned on the subject, and those who needed it have been revaccinated. To nurses in country districts and private institutions the warning may well be passed on, for truly it is the duty of every nurse to take this simple precaution against infection.

CONVALESCENT CHILDREN.—The preparations for the opening of country homes are going on apace, and Miss Anderson, the late matron of the Children's Hospital, Paddington Green, hopes shortly to be in charge of a convalescent home for her small charges who are returning to health. Ever since her accident two years ago Miss Anderson has been unable to fulfil all her former valuable services, and now seeks an easier sphere of work. Last year she had a cottage at Edgware for eight weeks for the convalescent children, but that was only a temporary arrangement. Everyone with hospital experience knows how difficult it is to secure change of air for children with abscesses or wounds; and how impossible it is to get any ordinary home to take in spinal cases for the three or six months' fresh air which can alone do them permanent good. It is to this work Miss Anderson has put her hand, and we wish her every success.

THE WOMEN'S INTERNATIONAL COUNCIL.—A great conference of women has lately been held at Washington, of which Mrs. Fawcett was president. Hundreds of women of all nations met daily for a week, and discussed the various problems of women and work. The subject of nursing received rather scant attention, the larger field of medicine being greatly to the fore. Yet several lady doctors in America have found their profession so precarious or its duties so harassing that they have been glad to retire to the quieter and safer work of nursing. It would be interesting to know if an ex-doctor makes a good nurse, or if her larger knowledge entices her beyond her province.

BLACKMAILING BY NURSES.—There has been some correspondence lately in the hospital papers on the subject of hospital and infirmary nurses exacting fees from the patients under their care as the price of proper attention. As usual, the accuser does not sign his name nor give particulars as to which institutions are supposed to be guilty of this disgraceful practice. The charge is one which is brought against nurses at intervals, and possibly has some foundation in isolated instances; but it is very unfair to make a sweeping accusation against the whole body of nurses in consequence. The sufferer should have the courage of his opinions and boldly complain to those in authority at the institution where the fault occurred. Every year our nurses are rising to a higher and nobler ideal, and no greater kindness can be done to them than to expose the failings of the lesser lights of the profession, that they may purge their ranks from such offenders.

A WORD OF ADVICE.—When a nurse leaves the hospital or institution where she has been trained, she should always leave her address with the matron or superintendent. Very often when a matron is asked to recommend a nurse to a vacant post she can remember several women she has trained who would be suitable, but they have left the institution and she does not know where to write to them. That they are seeking such situations she knows from various applications for their testimonials; but with the best wish in the world to help them she is unable to do so. Every nurse, therefore, should leave a permanent address with the head of her training school if she wishes to receive rapid promotion in her profession.

PROBATIONERS.—Our remark two weeks ago, that a probationer gets £10 or £12 a-year for being of very little use, seems to have given offence. Of course probationers do a certain amount of hard work—work which seems specially hard to those who were formerly unaccustomed to menial tasks; but so far as skilled nursing is concerned, they are generally untrustworthy. It is only natural; everything under the sun has to be learnt, and skill and competence come to no woman without long and patient experience of the subject in hand. Probationers must remember that in nearly every profession it is the rule for the learner to pay a premium for the privilege of being taught, besides giving his services during such time. In America it is common for nurses not only to receive no salary for the first year, but also to pay a small sum for their maintenance. We certainly think that the duties of probationers, which are arduous but not expert, are adequately remunerated by the small sum they receive and their board and lodging. Once they have passed their period of training and become certificated nurses, fit to be trusted with the care of the sick and dying, it is another thing. We do not think that nurses, as a rule, receive a high enough salary, but it is a question of supply and demand, and the thoroughly efficient nurse generally manages to push herself to the front and secure a lucrative post. The probationers need not be hurt if their duties are considered comparatively trivial, for once they blossom forth into charge nurses their responsibility and power in the greatest crises of life will be undeniable, and we would be the last to underrate them.

EARLY EFFORTS.—Why is extract of belladonna like a good lecture? Because it enlarges the capacity of the pupil.

Why is a standard taken in battle like marasmus? Because it is a-trophy.

Why is adhesion like a hasty marriage? Because it is union by first intention.

Why is a granulating sore like Brentford? Because it is near Ealing.—*The Lancet*, March 17th, 1827.

### The National Pension Fund for Nurses.

All Communications should be addressed The Editor, The Lodge, Porchester Square, W.

### WHAT THE NURSES ARE SAYING!

I had hoped some more competent person would have written on the article in the *Lancet* of April 21st. The writer seems writhing under the pains of The Hospital—I almost said agony. He really seems very ill over this Pension Fund; I think we nurses must take him in hand and nurse him. I am afraid the Pension Fund is turning his brain, and we can't afford to lose him. I have taken the *Lancet* for twenty years, and it has never been so much service to me as now, for I had written the B.N.A. for a form to join their ranks, when I saw the *Lancet* mourning over the Pension Fund in The Hospital. I had not heard of either, so I got my bookseller to get me The Hospital, and now I have judged for myself and joined the National Pension Fund. So you see I owe it a debt of gratitude. The *Lancet* has not always taken nurses to its bosom as now—in fact I have always thought the contrary, and such a lame objection to the Fund to say the ladies who had taken to nursing as a profession were affronted because they are taking to it as a pastime. Are the nurses who have and are bearing the burdens of the day to be losers by them? I know a good few county ladies who have taken to nursing—some because they can't get on at home. They go for a few years, then tire, give it up, some join the sisterhood and only work six or nine months in the year at most—in fact they generally take it very easy. There are a few exceptions, but so very few, and indeed these ladies don't always make the kindest of nurses.

As to thinking of the Fund as a charity, they do not know what they may need. Did the Irish sufferers think twenty years' ago that they would now be taking charity (real charity I am sorry to say)? Did the sufferers from the Glasgow City Bank think at one time they would now be living on charity?

I wonder what the *Lancet* would say if a number of gentlemen of means took to medicine to work for the love of the profession? It would soon raise a cry. I don't see why nursing should be chosen, of all the professions, to be followed for nothing; they might choose their favours more equally, and try medicine, law, and the Church, teaching, mothers' helps, &c. I am sure there would not be any trouble in getting thousands of nurses to join if they only knew about it. I think every hospital in the kingdom should be told to let their nurses know. I have known many a sad case of nurses. I hope the *Lancet* will receive the grateful thanks of Sister Christian.

### SOBER SENSE!

Will you please put my name on the list for N.P.F., as I cannot see how one is to lose their honest independence by paying towards a provision for old age. Those ladies who feel affronted have a remedy in their own hands. If they leave the field clear for nurses who are obliged to work for a livelihood, we shall have more chance of helping our broken-down fellow nurses to rest and change. I appreciate the kindness that promoted the scheme, and wish it all success.

Eastry, Sandwich. J. E. Lanham.

### HONEST DIFFICULTIES!

I have eagerly read all I have seen published in reference to the Nurses Pension Fund. It is in my opinion an excellent and much-needed fund, thanks to our kind benefactors for being the means of its existence. There would, I feel sure, be no difficulty in its progress, were we paid as is supposed in your issue April 7th, viz., a nurse 25 years of age getting £25 per annum. In several hospitals I know a candidate is not accepted as probationer under 25. She will then get £8 or £10 for first year, £14 or £15 for second; but must at least have been in the hospital four years before she earns £25 per annum. So to get a pension of £15 a-year at 50 years of age (according to Table II.), instead of £1 18s. being the quarterly contribution out of £25 per annum, it really is £2 8*s*. 8*d*. This leaves a salary of £15 5*s*. 4*d*., which I think you will agree with me in thinking scarcely sufficient for ordinary expenditure, especially when we have no outdoor uniform found. Now I have tried to take an example as reasonable as possible; my own is (and I know lots the same) a far worse case. I have been four years in an hospital, am 32 years of age, getting £21 per annum, and though very wishful to join the Pension Fund, cannot do so out of my salary. Wishing success to those who can.

A Nurse.

The case of our correspondent is exceptional. Her course is to apply to the Secretary, 38, Old Jewry, E.C., at once, for a special form, when most if not all of her difficulties will be removed.

### THE NATIONAL PENSION FUND FOR NURSES.

(From the *British Medical Journal*, May 5th, 1888.)

Sir,—Now that the subject of the Pension Fund is under discussion, there are many nurses who would be glad to have their own opinion on the matter made public, and who would be grateful to you if you would make it known through your paper. In the first place, surely the word "pension" is misleading; the dictionaries define it as "an allowance made without an equivalent." Of course, no nurse would be so unreasonable as to expect her services to be so rewarded, but there certainly is a very widely spread feeling of disappointment that the terms of the Pension Fund are such as to render it almost an impossibility for nurses to join it.

So much has been written in The Hospital about the "duty of making provision for the noble women who are devoting their lives to the nursing of the sick," that the first feeling of receiving the prospectus was one of surprise that the provision was to be made almost entirely by themselves. It is true that very young nurses will find the terms comparatively easy, but the vast number, who have already spent many years of their lives in this work, will find it difficult, if not impossible, to spare regularly from £20 to £40 per annum out of a salary which, in the case of nurses, never exceeds £40, and is in most instances considerably less.—I am, etc., A Nurse.

On this letter the Editor of the *British Medical Journal* makes the following remarks:—

"We believe that self-help and self-respect will be found to be the dominant principles by which t e great body of nurses are animated. At the discussion of the Society of Arts in October last, when the Fund was first instituted, the founder emphatically stated that the Pension Fund was one of providence, and was not eleemosynary. Nurses were then, and are now, wanted to join the fund by paying into it at least one-eighth of their earnings year by year. All who do this may, we believe, rest assured that those who have already shown so much liberality and interest in the cause of nurses by promoting this fund will take steps to conserve the best interests of every nurse who entrusts her savings to their care. There are necessarily a large number of nurses who have already devoted many years of their lives to nursing, and who have no reserve, who will, as our correspondent points out, 'find it difficult, if not impossible, to spare regularly from £20 to £40 per annum out of a salary which, in the case of nurses, never exceeds £40, and is in most instances considerably less.' The managers of the fund, with the object of meeting such cases, we believe notified that nurses so placed, and who are desirous of being assisted to help themselves, should apply to the Secretary, 38, Old Jewry, E.C., for a special form of application to be filled up and returned with as little delay as possible. With reference to some statements which have been published, and are being circulated, intimating that the payments required are from an actuarial point of view excessive, it is right to say that they are effectually refuted by the report of Mr. King, the well-known actuary, and that the comparisons therein made are based on palpable error."

### AN EXAMPLE TO BE FOLLOWED.

We note in the *Portsmouth Times* a letter signed W. H. Axford, which gives an interesting and lucid explanation of the method and rates of the National Pension Fund for Nurses. As there are doubtless many nurses who would join the Fund who are not acquainted with the details, or cannot understand very easily the tables in which these are set forth, we wish that many people would follow Mr. Axford's example and write to the local newspapers, explaining the meaning and method of the Pension Fund where it will come under the eyes of the nurses of the neighbourhood.

### A WORD TO THE OLDER NURSES.

Many nurses have expressed themselves as terribly disappointed at not being able to join the Pension Fund, owing to the high rates of premium which they would have to pay to secure a minimum pension of £30 per annum. A nurse 30 years of age, who has spent the best years of her life in nursing, necessarily finds it impossible to pay £33 per annum to secure such a pension at 50 years of age. They very reasonably point out that from a business point of view such a payment is no doubt necessary to secure the retiring allowance at 50, which they desire, seeing that one can buy an annuity as one can buy clothes or books—that is, at a fixed price for a certain article. It affords us great pleasure to assure all nurses similarly circumstanced to her whose case we here quote, that the Pension Fund is not entirely a matter of business, and that those who have established it have determined to make it a real help to all nurses who will exercise sufficient self-denial to pay into the fund at least one-eighth of their earnings year by year. The spontaneous gift of £25,000 should give all nurses confidence in those who have the management of the National Pension Fund, and should make every nurse determine to apply for a special form, and to contribute at least one-eighth of her income on the returnable scale. By so doing she will help her best friends to help herself to an extent adequate to provide for her needs in the day of her necessity. Every day nurses are joining the fund, and the concession of the one-eighth scale should induce every sensible nurse to procure and fill up a "special form" forthwith.

### Appointments.

Miss Mary Lambert has been appointed matron of the Southport Infirmary. Miss Lambert was trained at Grimsby Hospital upwards of eight years ago, and afterwards had charge of various wards at the North Riding Infirmary. For the last three years Miss Lambert has held the post of matron at the Carmarthen Infirmary, and her departure to fresh fields is much regretted by the medical staff and committee.

### Exchange and Mart.

Under this heading we propose to insert purely Exchange advertisements, at a charge of sixpence for eighteen words, or three insertions for one shilling It must be distinctly understood that ordinary advertisements will not be inserted here.

### WELL-DESERVED CHASTISEMENT.

It is by a spirit of poetic justice that fate has decreed that the present editors should also be the proprietors of the *Lancet* newspaper. Were it otherwise the conduct of that journal of late would have speedily brought about a change of editorship. The latest, if not the most flagrant, example of the abuse to which it is possible to put the trust imposed upon an editor by the influence of a great journal is the conduct of the editors of the *Lancet* towards the actuary of the National Pension Fund.

Mr. George King, as a professional man of the highest standing, had the right to expect fair treatment at any rate. The treatment he has received is explained by him in the postscript to his report, dated the 1st instant, which is now before us. It runs as follows: "In his article of the 28th April, the editor of the *Lancet* challenged me to call his figures in question, and the above report is my reply. The editor, however, with his usual courtesy, has refused the time necessary for the answer to reach him, and has hastened to reprint his articles in pamphlet form and to circulate them broadcast among all the hospitals in the kingdom. Is it possible to find parliamentary language which will properly characterise such conduct?"

The *Lancet* attacks Mr. King's figures on two points, which we will print in italics, giving Mr. King's reply in ordinary type.

I. The *Lancet* states: "*So far is the Post Office Department from representing the open market in this matter, that in his last report the Postmaster-General returned the new business of this class last year as* 87 *annuities amounting to* £1,772, *and the total business on his books as comprising* 857 *annuities of this description.*"

Mr. King replies: "Even when the editor of the *Lancet* professes to quote statistics exactly, he blunders hopelessly. Return No. 99, on Government Insurances and Annuities, ordered by the House of Commons to be printed on the 5th of April last, shows that the Government new business for 1887 consisted of 100 deferred annuities for £2,049, and at the close of the year there existed 1,155 deferred annuities for £26,312. It is perhaps hardly necessary to remark here that the National Debt Office and the Post Office form only one establishment, in so far as the grant of deferred annuities is concerned." The object of the *Lancet* in quoting the figures was to show that the Government Department does not really rule the market, and the impression desired to be conveyed was that the business of the insurance companies is enormously larger than that of the Government. Mr. King proves that "the business of all the British offices is barely more than one-third of the business of the Government."

II. The *Lancet* next quotes from the prospectus of three companies to show that the rates of the National Pension Fund are excessive. The benefit selected *is a* "*deferred annuity of* £15 *per annum to be granted to an entrant in the twenty-fifth year of age, and enjoyed after the attainment of sixty years. The annual premium, as tabulated, is as follows: Gresham,* £2 0*s.* 2*d.*; *Star,* £2 6*s.* 8*d.*; *Kent,* £1 17*s.* 9*d.*; *Nurses Pension Fund,* £2 10*s. Will the actuary venture to call these figures in question or to publish a further correction?*"

Will it be believed that the above Insurance Companies' rates are for male lives, those for the Pension Fund being for female lives, and the male rate of the Pension Fund being £2 2*s.*, not £2 10*s.*, as compared with £2 6*s.* 8*d.* charged by the Star Office. We must leave Mr. King's report to deal with the Gresham, as we have not space to quote at length; and as there is no life office called the Kent—although there is an United Kent Life Office which does not transact annuity business—nothing more need be said about it.

We wish we had space to quote the whole of Mr. King's report, as the chastisement he gives to the conductors of the *Lancet* is so severe, although couched in moderate terms, that we are positively sorry for the loss of prestige which must result to our old but degenerate contemporary. Why it should have gone out of its way to attack an altogether worthy and much-needed undertaking like the National Pension Fund for Nurses it is not easy to imagine.

At any rate, it is to be hoped that the editors will leave figures for the future to those who understand them, and stick to medicine and science, which leaves a wider margin for inaccurate declamation, as their conclusions have never been regarded as all precisely demonstrable.

### Everybody's Opinion.

A RULE against the giving of presents to nurses by grateful patients may be only right in your opinion, but in my humble estimation it is also very unpleasant. The institution to which I have the honour to belong has a rule, recommending employers "not to offer any gratuity to a nurse." This, I think, goes quite far enough, and spares the feelings of all concerned. A rigid rule against gratuities very naturally suggests the inquiry, What on earth would these nurses expect if only they were allowed to have expectations? It savours rather too much of the "followers not allowed" order of things. I cannot pity anyone who is in great perplexity as to what a nurse shall expect at their hands. We hope to deserve esteem, respect, and a moderate amount of kindness, and these often fall to our lot. We are paid by rule—eat, sleep, work, drink by rule; therefore friendships, presents, what you will, if they concern nurses, let us have a rule.

W. H.

### "A PROBATIONER'S PROTEST."

I HAVE been a probationer in two hospitals—in one for twelve months and in the other for eighteen months—and I can conscientiously maintain that the nursing in those two hospitals was either done personally or under the supervision of the charge nurse, who, instead of being task-mistresses, gather the straw and assist in making the bricks, by teaching me to do dressings and nursing in general, which the so-called task-mistresses could have done in half the time. Nurse V. forgets to acknowledge the charge nurses bear the blame of any neglected or ill-done work. The staffs visit on them the mistakes committed by the probationers, as well as giving them the credit of the latter's cleverness.

Bristol. NURSE P.

I read in your "Nursing Mirror" that at some institutions nurses are paid a percentage on their earnings over a fixed salary. In my opinion, all institutions, and the public, would be benefited by institutions paying their nurses a fixed salary and a certain sum per week for every week they are employed, and for the amount per week to increase each year. I should be glad of the opinion of others on this matter, for the true nurse would also be considerably benefited by this change.

H. J., Matron.

### Vacancies.

The following vacancies are announced :—

**Matrons.**

Hangleton Hospital, Hove.

Atkinson Morley's Convalescent Hospital.

Blackheath Cottage Hospital.

Lying-in Hospital, Liverpool.

Carmarthenshire Infirmary.

**Nurses.**

Shoreditch Infirmary; £20.

Dewsbury Infirmary; £20.

Hospital for Chest Diseases, City Road; £22

Stockport Infirmary, £25.

Hospital of St. Cross, Rugby; £25.

Blackheath Cottage Hospital.

Newark Hospital Nurses' Institute; £20.

Eye and Ear Hospital, Bradford.

Union Infirmary, Everton, Liverpool; £18.

Edinburgh Poorhouse; £30

Edinburgh Hospital; £25.

Birkdale Home, Southport.

Victoria Home, Bournemouth.

Cornwall Trained Nurses' Home, Truro.

Kingston Nurses' Home, Baker Street, Hull.

**Probationers.**

Children's Hospital, Bristol.

District Infirmary, Dewsbury.

Home and Infirmary for Sick Children, Lower Sydenham.

Grimsby Hospital, Lincolnshire.

Denbighshire Infirmary; £18.

Lowestoft Hospital.

Workhouse Infirmary Nursing Association, 6, Adam Street, Strand.

**District Nurses.**

Chester Diocesan Deaconess Institution. Slough.

**Attendants on Insane.**

Fulham Union; £20.

The Coppice, Nottingham, £4.

### Anecdotes of Personal Adventure.

HAPPILY! A POPULAR DELUSION.—How difficult it is to convince patients' friends that a nurse is not, by virtue of her profession, given to indulgence in stimulants may be seen from the two following anecdotes. I was sent to nurse a young man suffering from pneumonia, in the north of England. The first day I was there a bottle of port wine and a tumbler was sent up with my dinner to the room adjoining my patient's. On my telling the patient's mother that I was an abstainer, she quietly put the bottle and glass in a corner of the room out of sight, and said she would leave it there as I might like to have some when alone. She evidently thought that I, like Mrs. Gamp, would like to be able to "put my lips to it when so dispoged." Again, I was in charge of a women's ward in a county hospital. One visiting afternoon, when one of the patient's friends was speaking to me about her, I felt something hard slipped into my apron pocket, and a voice said in a stage whisper, "A drop of something short to put in your tea." It is needless to say the bottle was immediately returned—I am afraid without thanks. Happily, I think the idea is fast dying away that the only way to ensure a nurse's attention is through her pocket or stomach.—*Nurse Nancy.*

AN AWKWARD DILEMMA.—"A many years ago, when I was young and charming," or, at any rate, slender, and ready for anything in the shape of fun that turned up, I happened to be with another probationer as young, slender, and giddy as myself, in one of the small rooms in the hospital laundry. The laundress picked up the matron's corsets, which had just been washed, and showed them to us. They were of the old-fashioned kind—no fasteners in front and laced behind. Now our matron was "fat, fair, and forty." My friend said, "Just look at the size of them?" I exclaimed, "Why, we could both get inside them." No sooner said than done. We embraced each other, and the laundress laced us up. Just as she had finished we heard the matron's voice, and the laundress had to leave us, like the Siamese Twins or an artificial two-headed nightingale, inseparably united, and go round with her. We broke out into a cold perspiration, fearing every moment that the matron might appear, and then what consequences might follow! At last the laundress returned—thank goodness, alone—and released us from durance vile. Dear old mater! I hope she never knew of our thoughtless trick. We would not have hurt her feelings on any account. She has long since joined the majority, and I, alas! (there is a moral to this tale, be it known) am now a matron weighing fourteen stone myself! I wonder what has become of my partner in mischief! If she reads these lines she will, I am sure, have a hearty laugh at the remembrance of our escapade.

A. E. B.

### Notes and Queries.

TO CORRESPONDENTS.—1. Questions may be written on post-cards. 2. Advertisements in disguise are inadmissible. 3. In answering a query please quote the number. 4. If a private answer is desired, a stamped addressed envelope must be forwarded.

(12) *Erin* would be glad to know if there is any place in London where she can learn dispensing at a moderate charge, and without giving up all her time to it.

(13) *H.H.P.* would like to hear of a home in the midland counties where a boy of 15 with fractured spine and complete paralysis of the lower extremities could be received for a small monthly payment.

(14) Do any of our readers subscribe to the Domestic Servants' Benevolent Institution? If so, would they please vote for Eliza Plumb.

**Answers.**

(11) Children's Hospitals take probationers of 21, but there is usually a long time to wait for vacancies "Probationer" is advised to apply to country infirmaries.

*H.H.P.*—There is no connexion whatever between the two schemes.

*Mrs. Browne* is referred to the note "Cottage Nurses" in our last number.

